# Appropriateness of Intrapartum Antibiotic Prophylaxis to Prevent Neonatal Group B Streptococcus Disease

**DOI:** 10.1371/journal.pone.0166179

**Published:** 2016-11-18

**Authors:** Aida Bianco, Elisabetta Larosa, Claudia Pileggi, Maria Pavia, The Collaborative Working Group

**Affiliations:** Department of Health Sciences, University of Catanzaro "Magna Græcia", Catanzaro, Italy; Centre Hospitalier Universitaire Vaudois, FRANCE

## Abstract

The aims of this study were to describe the adherence to CDC guidelines for intrapartum antibiotic prophylaxis (IAP) and to identify possible factors influencing noncompliance with guidelines. We conducted a retrospective study in Italy. Our cohort included women in whom antenatal Group B *Streptococcus* (GBS) screening was not performed, was performed, but results were not available at the time of labor or delivery and women who were positive for GBS colonization. The indications for complete execution of IAP according to revised CDC guidelines was evaluated. It was considered adequate when performed with a recommended antibiotic at least four hours prior to delivery. The cohort included 902 women. Among those who had performed rectal and vaginal swabs (or recto-vaginal swabs), results were available in 86.9% of vaginal swabs and in 87.1% of rectal swabs and GBS was detected in 59.8% of vaginal swabs and in 71% of rectal swabs. 49.2% women had indication for GBS prophylaxis. Among these, 91.1% received an antibiotic during labor. Totally appropriate IAP was performed in 36.3% deliveries, an inappropriate antibiotic was administered in 10.4% women, the remaining 45.3% women received partially appropriate IAP; of these, 15.5% had received antibiotics through an inappropriate route of administration, 18.2% an inappropriate dosage regimen. Overall, 27.5% women received intrapartum ampicillin with inappropriate timing. Multivariate analysis showed that totally appropriate prophylaxis was significantly more likely in women who had no previous live birth, who had vaginal delivery, and a positive result at antenatal GBS screening. Despite satisfactory GBS screening implementation, there is still a substantial gap between optimal and actual IAP. We hypothesize that the complexity of the CDC guidelines may partially explain this shortcoming. Future efforts will include initiatives focused at enabling and reinforcing adherence to evidence-based prevention practices.

## Introduction

Early-onset group B streptococcal disease (EOGBSD) has long been the major infectious cause of first-week neonatal morbidity and mortality [1] and infants born to heavily recto-vaginal Group B Streptococcus (GBS) colonized women have been found to be at an increased risk of neonatal sepsis [2]. Epidemiological studies have revealed that pregnant women colonized with GBS are 25 times more likely to deliver infants developing EOGBSD than women with negative prenatal cultures [3,4]. Maternal intrapartum antibiotic prophylaxis (IAP) rather than treatment is the most effective means to reduce neonatal GBS infections and the burden of the disease. Current CDC recommendations promote culture-based screening of all pregnant women at 35–37 weeks of gestation and IAP for GBS-positive women [5]. The 2010 Italian Guidelines on Physiologic Pregnancy (revised in 2011 by the National System for Guidelines) [6] are totally in agreement with the CDC guidelines. As a result of these guidelines, the overall incidence of EOGBSD has progressively declined in countries or regions that have introduced routine screening and IAP [7,8].

Few previous studies have analyzed IAP appropriateness and its overall adherence to recommended guidelines, ranging from 50% to 71% [9–12].

The aims of this study were to explore to what extent pregnant women undergo GBS screening and, among those with an indication for IAP, to evaluate the adherence to CDC guidelines [5] to prevent EOGBSD and to identify possible factors influencing noncompliance with guidelines.

## Materials and Methods

We conducted a retrospective study at 4 randomly selected delivery units in Calabria, Italy, from January through December 2014.

From the total cohort of pregnant women, we first excluded those who: 1) were <18 years at delivery and 2) delivered infants with a diagnosis of intrauterine unexplained fetal death. Only one twin pregnancy was accounted for. Since our aim was to describe the adherence to IAP according to revised CDC guidelines, we excluded women with documented negative results at GBS antenatal screening. Therefore, our cohort included: 1) women in whom antenatal GBS screening was not performed; 2) women in whom screening was performed, but results were not available at the time of labor or delivery; 3) women who were positive for GBS colonization.

Maternal and obstetrical data, and bacterial culture for GBS results were obtained from medical records. The information was extracted by two trained physicians, having experience in clinical documentation. The information included sociodemographic data (age at delivery, marital status, education level, working activity, nationality), documented beta-lactams allergy, gestational age at birth, labor onset, mode, date and hour of delivery, antenatal GBS screening and culture results, risk factors for GBS infection (maternal colonization with GBS in the genitourinary or gastrointestinal tracts, GBS bacteriuria at any time during current pregnancy, positive anamnesis for previous infant with diagnosis of early-onset GBS disease), risk factors of early-onset GBS disease (intrapartum maternal temperature >38°C, amniotic membrane rupture ≥18 hours, < 37 gestational weeks at delivery), details of intrapartum chemoprophylaxis (choice of antibiotic, route and time of drug administration, dosage regimen). We then evaluated the indications for complete execution of intra-partum antibiotic prophylaxis (IAP), that was considered adequate when performed with a recommended antibiotic at least four hours prior to delivery [13].

According to revised CDC guidelines for the prevention of neonatal GBS disease [5], all pregnant women should be screened at 35–37 weeks’ gestation for vaginal and rectal GBS colonization. IAP administration, at the time of labor or rupture of membranes, should be performed in several cases: 1) women who tested positive for GBS colonization, except those with a planned caesarean section before onset of labor and with intact amniotic membranes; 2) women in whom screening was not performed or results were not available at the time of labor or delivery, and presented at least one of any following conditions: a) women who were <37 weeks and 0 days’ gestation; b) who had a duration of membrane rupture ≥18 hours; c) who had a temperature of ≥38°C.

Moreover, IAP is indicated in: 1) women positive for GBS isolated from the urine at any time; 2) women with symptomatic or asymptomatic GBS urinary tract infection detected during their current pregnancy; 3) women who had a previous infant with invasive GBS disease.

Penicillin is the recommended IAP agent but, as it is not produced in Italy, ampicillin is routinely administered as a standard dose of 2 g intravenously from the onset of labor plus 1 g intravenously every 4 hours until delivery. Beta-lactam allergic patients receive erythromycin or clindamycin intravenously in equivalent dosage. Women with reported beta-lactam allergy, but at low risk for anaphylaxis should receive cephazolin, while those at high risk of anaphylaxis (prior history of anaphylaxis, angioedema, respiratory distress or urticaria following administration of a penicillin or cephalosporin) should receive clindamycin (if the GBS is susceptible) or vancomycin.

The IAP has been judged totally appropriate if all items were in accordance with the guidelines, partially appropriate if antibiotic was chosen from preferred or alternative antibiotics suggested by the guidelines, but at least one of the other main components did not follow adherence to the guidelines, and inappropriate if the antibiotic choice was not recommended by the guidelines or all four criteria were considered as non-compliant with the guidelines.

Approval from the Institutional Ethics Committee (“Mater Domini” Hospital of Catanzaro, Italy) was obtained (10/03/2015) As a matter of course, written informed consent is always requested when admission to the delivery units occurs, and only the patients who had given permission for their personal data to be used for research were included in the study.

### Statistical analysis

Univariate analysis and multivariate stepwise logistic regression analysis were performed. Univariate analysis was performed using Chi-square test. A model was developed including those variables potentially associated with the following outcome of interest: IAP appropriateness (0 = not appropriate/partially appropriate, 1 = totally appropriate) (Model 1), according to CDC guidelines. Model building strategy and particularly ways to include independent variables in the model (ordinal or categorical) took into account how each of these ways better fitted the data at the univariate analysis and we chose that way in the multivariate analysis. Independent variables for which p was <0.25 in univariate analysis were included in the multivariate models. In the model the explanatory variables included were the following: nationality (0 = Italian, 1 = other), previous live birth (0 = none, 1 = 1 or more), type of birth (0 = normal spontaneous vaginal delivery, 1 = planned cesarean section, 2 = emergency cesarean section), antenatal GBS screening (0 = not performed, 1 = GBS positive, 2 = GBS unknown) included as a dummy variable with GBS positive being the reference category. Adjusted odds ratio (ORs) and 95% confidence intervals (CIs) were calculated. The significance level for variables entering the logistic regression models was set at 0.2 and for removal from the model at 0.4. A two-sided *p*-value of 0.05 or less was considered as indicating a statistically significant difference. The data were analyzed using the Stata software program, version 14 [14].

## Results

**Fig 1:**
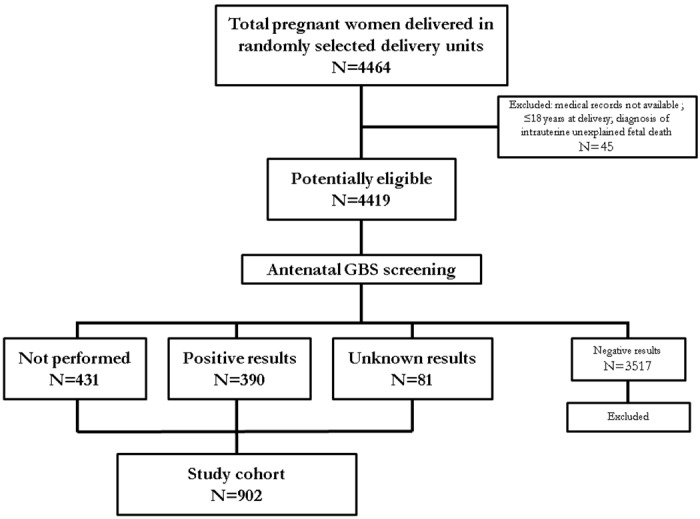
Study population. This figure shows population of women who delivered in selected maternity units and overall study cohort.

During the study period a total of 4464 women delivered in maternity units of four selected hospitals. Of these, 45 were excluded because: 1) medical records were not available; 2) women were ≤18 years at delivery; 3) intrauterine unexplained fetal death was detected. Of the remaining 4419, 3988 (90.2%) were examined with GBS antenatal screening (rectal or vaginal swab or both). Among eligible women who had performed GBS antenatal screening, 3517 (88.2%) showed negative culture results and thus were excluded from the cohort. Of the remaining, 390 (9.8%) resulted positive to GBS screening and 81 (2%) performed GBS antenatal screening with unknown results at the time of labor or delivery. Only 431 (9.8%) of all eligible women did not undergo GBS antenatal screening. Therefore, the overall cohort included 902 women (Fig 1).

The mean age of the study population was 31.3 years with an age range between 18 and 54 years. The vast majority (80.5%) were married and 386 (44.1%) had completed high school. 719 (79.7%) were Italians and about 46% were housewives. More than half of the sample (58.7%) had spontaneous vaginal deliveries, and 164 (18.2%) had emergency cesarean sections. The mean gestational age at birth was 38.1 weeks (±2.7 SD). Overall, 429 (47.6%) of the women were primigravidas and multiple births occurred in 19 (2.1%). For women whose gestational age at the prenatal cultures were recorded, 70 (25.2%) were at <35 weeks gestation, 173 (62.2%) at 35–37 weeks, and 35 (12.6%) at >37 weeks; among these, 18 (6.5%) were cultured as soon as possible after hospital admission, in emergency. Among women of the overall cohort, 471 performed antenatal GBS screening, culture sites were rectal and vaginal (or recto-vaginal) in 435 (92.4%) women; the remaining cultures were collected at the vaginal site in only 35 (7.4%) women and at the rectal site in only 1 (0.2%) woman. Among those who had performed rectal and vaginal swabs (or recto-vaginal swabs), results were available in 378 (86.9%) of vaginal swabs and in 379 (87.1%) of rectal swabs and GBS was detected in 260 (59.8%) of vaginal swabs and in 309 (71%) of rectal swabs. The antimicrobial sensitivity pattern of isolates was available for only 11.1% of positive swabs, and in 63.6% of these tetracycline-resistant strains were observed. The prevalence of self-reported beta-lactam antibiotic allergy was 36 (4.1%).

**Fig 2:**
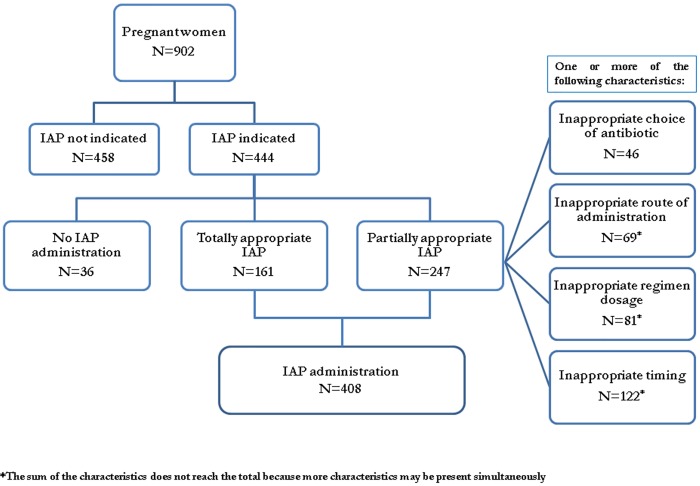
IAP administration approach. This figure reports women who had indication for GBS chemoprophylaxis among study cohort and intrapartum antibiotic prophylaxis administration approach.

Among the study cohort, 444 (49.2%) women had indication for GBS chemoprophylaxis (Fig 2). According to CDC guidelines, the main reasons to indicate IAP administration were: documented positive results at antenatal GBS screening in 308 women; unknown GBS status (not performed or undocumented results of antenatal GBS screening) at the onset of labor in the presence of the following risk conditions: i) preterm delivery (at <37 weeks and 0 days’ gestation) in 93 women; ii) duration of membrane rupture ≥18 hours in 26 women; iii) both previous risk conditions in 16 women; iv) observed temperature ≥38°C at delivery in 1 case. The IAP administration approach is reported in Fig 2. Among women with IAP indication, 408 (91.1%) received an antibiotic during labor. Totally appropriate IAP was performed in 161 (36.3%) deliveries, an inappropriate antibiotic was administered in 46 (10.4%) women, the remaining 201 (45.3%) women received partially appropriate IAP; of these, 69 (15.5%) had received antibiotics through an inappropriate route of administration (intramuscular or oral) and 81 (18.2%) an inappropriate dosage regimen. Overall, 122 (27.5%) women received intrapartum ampicillin with inappropriate timing. Among those who declared a history of allergy to beta-lactam antibiotics, two were treated with clindamycin (but no susceptibility testing was performed prior to administration, despite it was recommended) and vancomycin, respectively.

Appropriateness of IAP according to various characteristics of pregnant women and results of univariate analysis are is illustrated in Table 1. The univariate analysis showed that totally appropriate IAP administration was significantly more likely in women who delivered at ≥37 weeks gestation (χ^2^ = 17.17, p<0.001) compared with those whose delivered preterm (41.8% vs 20.1%), who had no previous live birth (χ^2^ = 15.37, p<0.001) compared with those had one or more previous live birth (44.2% vs 26.2%), who had vaginal delivery (χ^2^ = 19.09, p<0.001) compared with those had emergency cesarean section (42.7% vs 20.8%), and a positive result at antenatal GBS screening (χ^2^ = 27.39, p<0.001) compared with those whose results were unknown or who had not performed screening (44.2% vs 31.7%). Moreover, totally appropriate IAP administration was significantly higher in Italian pregnant women (χ^2^ = 6.89, p = 0.009) compared with foreign individuals (38.9% vs 22.5%). Results of the multivariate stepwise logistic regression analysis substantially confirmed those of the univariate analysis except for gestational age that was removed from the model (Table 2).

**Table 1:** Appropriateness of IAP according to several characteristics of pregnant women.

		All		IAP indication				
				Total		Totally appropriate administration*		p
		*N*	*%*	*N*	*%*	*N*	*%*	
**Age, years**								
	*<25*	*131*	*14*.*5*	*60*	*13*.*5*	*21*	*35*	*0*.*651*
	*25–29*	*218*	*24*.*2*	*107*	*24*.*1*	*45*	*42*.*1*	
	*30–34*	*274*	*30*.*4*	*142*	*32*	*50*	*35*.*2*	
	*35–39*	*206*	*22*.*8*	*101*	*22*.*7*	*35*	*34*.*6*	
	*≥40*	*73*	*8*.*1*	*34*	*7*.*7*	*10*	*29*.*4*	
**Education level**†								
	*No formal education/Completing primary school*	*84*	*9*.*6*	*33*	*7*.*7*	*9*	*27*.*3*	*0*.*279*
	*Completing secondary school*	*211*	*24*.*1*	*83*	*19*.*3*	*24*	*28*.*9*	
	*Completing high school*	*386*	*44*.*1*	*200*	*46*.*5*	*76*	*38*	
	*Holding a bachelor’s degree or any college degree*	*195*	*22*.*2*	*114*	*26*.*5*	*45*	*39*.*5*	
**Nationality**								
	*Italian*	*719*	*79*.*7*	*373*	*84*	*145*	*38*.*9*	*0*.*009*
	*Other*	*183*	*20*.*3*	*71*	*16*	*16*	*22*.*5*	
**Gestational age, week**								
	*<37 weeks of gestation*	*198*	*22*	*114*	*25*.*7*	*23*	*20*.*1*	*<0*.*001*
	*≥37 weeks of gestation*	*704*	*78*	*330*	*74*.*3*	*138*	*41*.*8*	
**Previous live birth**								
	*None*	*429*	*47*.*6*	*249*	*56*.*1*	*110*	*44*.*2*	*<0*.*001*
	*1 or more*	*473*	*52*.*4*	*195*	*43*.*9*	*51*	*26*.*2*	
**Course of pregnancy**								
	*Physiologic*	*619*	*68*.*9*	*323*	*72*.*7*	*121*	*37*.*5*	*0*.*390*
	*Pathologic*	*283*	*31*.*4*	*121*	*27*.*3*	*40*	*33*.*1*	
**Type of delivery**								
	*Normal spontaneous vaginal delivery*	*530*	*58*.*8*	*314*	*70*.*7*	*134*	*42*.*7*	*<0*.*001*
	*Planned cesarean section*	*208*	*23*		*-*	*-*	*-*	
	*Emergency cesarean section*	*164*	*18*.*2*	*130*	*29*.*3*	*27*	*20*.*8*	
**Antenatal GBS screening**								
	*Not performed*	*431*	*47*.*8*	*120*		*23*	*19*.*2*	*<0*.*001*
	*GBS positive*	*390*	*43*.*2*	*308*		*136*	*44*.*2*	
	*GBS unknown*	*81*	*9*	*16*		*2*	*12*.*5*	
	**Total**	*902*		*444*	*49*.*2*	*161*	*36*.*3*	

* Totally appropriate administration: administration in accordance with all characteristics of the revised CDC guidelines^5^

^†^ Percentages are calculated on available data (876)

**Table 2:** Logistic regression model results on predictors of appropriate IAP administration.

Model 1. Outcome:		Totally appropriate IAP administration	
		Log-likelihood = -261.78, χ^2^ = 0.00, p<0.0001, No. of obs. = 444	
Variable			
		OR	95%CI
**Nationality**			
	*Italian*	*1*.*00*	
	*Other*	*0*.*53*	*0*.*28–0*.*99*
**Previous live birth**			
	*None*	*1*.*00*	
	*1 or more*	*0*.*41*	*0*.*27–0*.*63*
**Type of delivery**			
	*Normal vaginal delivery*	*1*.*00*	
	*Emergency cesarean section*	*0*.*68*	*0*.*52–0*.*88*
**Antenatal GBS screening**			
	*GBS positive*	*1*.*00*	
	*Not performed*	*0*.*41*	*0*.*24–0*.*71*
	*GBS unknown*	*0*.*23*	*0*.*05–1*.*06*

## Discussion

Our study provides a comprehensive assessment of antenatal GBS screening and an in depth evaluation of IAP administration in pregnant women in our area of investigation.

Introduction of recommended universal culture-based antenatal screening of all pregnant women at 35–37 weeks of gestation is likely to have contributed to the documented decline in the incidence of early-onset group B streptococcal (EOGBS) disease [15]. The data in our study confirm that antenatal GBS screening has been successfully implemented in our area of investigation. Indeed, 90.2% of women were examined with GBS antenatal screening and 62.2% at the appropriate gestational age. Moreover, we found an available documented result at delivery in 98% of the screened women with GBS antenatal screening. The percentage of GBS screened women was higher than the 85% reported from other national and international studies [12,15], and this increase in the adoption of screening has been probably coupled with the use of other recommended prenatal screening tests and with the greater prenatal care utilization [9]. GBS colonization was 9.8% in our population; which is lower than that reported in many previous studies in the literature [9,11,12,16,17], although it is well known that GBS maternal colonization varies from place to place. Moreover, other factors may have contributed to this variation, including socio-economic factors, variable clinical practices, methods in sample collection and processing techniques, as well as ethnic and genetic factors that may play a role in rates of GBS infection [18]. Although about two-third of participants had performed screening at the recommended time, our findings showed about 25% of women were tested before the recommended 35 weeks of gestation. Antenatal GBS screening outside the recommended window may yield false negative results, because GBS colonization during pregnancy can be transient [19]; furthermore, to improve culture test performance, CDC encourage screening less than 5 weeks before delivery [20].

As regards the choice of culture site, in women who were screened through both vaginal and rectal swabs, but separately, GBS was detected in 59.8% and in 71% of vaginal and rectal swabs, respectively, confirming that GBS colonization of rectal samples is higher than that of vaginal samples [21], and that rectovaginal sampling is more appropriate than vaginal sampling only [22–25].

Findings of our study showed that IAP was indicated in almost half (49.2%) of pregnant women and, among these, we report a high rate (91.1%) of IAP administration, in agreement with CDC revised guidelines. Similar results were observed in previous studies [15,26–28]. However, totally appropriate IAP, evaluated on all four criteria (drug choice, route of administration, dosage regimen and timing), was achieved in only 36.3% of pregnant women, and this low rate is a cause for concern, since missing or partially appropriate IAP may expose newborns to an increased risk of EOGBSD [29]. In previous studies, the most significant criterion adopted to evaluate IAP appropriateness was the timing, whereas other IAP items were not analyzed in detail. Therefore, comparisons with other studies should be made cautiously. Among women with an indication for GBS prophylaxis, Goins et al. [9] and De Luca et al. [11] reported optimal IAP in 61.2% and 50% of deliveries, respectively. Berardi et al. [12] in a prospective cohort study showed that adequate IAP was administered in 52% of GBS culture-positive women and in 62.2% of the women with unknown GBS status and risk factors.

Regarding the choice of drug, the reported practice was well aligned with the guidelines for patients with no allergy to penicillin. Since GBS strains have become increasingly resistant to most of the alternative agents, this particular clinical situation is one in which there is the potential for improvement in compliance with the guidelines [30].

Another opportunity for improvement is related to the timing of IAP administration [30]. Recent guidelines recommend IAP administration at least 4 hours before delivery: we reported that 27.5% of women received intrapartum ampicillin with inappropriate timing. Although health-care providers are unable to adhere to this recommendation in women who deliver precipitately and the most recent CDC guidelines state that no necessary obstetric procedure should be delayed to achieve 4 hours of GBS prophylaxis before delivery [5]. Therefore, the key question is whether newborns, delivered by women who had not received IAP at least 4 hours before delivery have any protection against vertical GBS transmission [31]. The origin of this four-hour timing criterium for IAP is unclear [10], but previous studies assessing the optimal timing of IAP for maternal colonization with GBS, without other risk factors, have shown increased rates of neonatal GBS colonization, but no increased risk of neonatal sepsis in infants whose mothers received antibiotic prophylaxis <4 hours before delivery [32–34].

In the present study type of delivery, availability and result of GBS screening and previous live births showed to be significantly associated to IAP appropriateness. The finding that vaginal delivery and a positive result to GBS screening were predictors of appropriate IAP may be related to a more straightforward guidelines description of IAP practice in these circumstances, whereas the guidelines algorithm explaining how to implement IAP in case of caesarian section and in presence of unknown results or not performed screening appear to be more complicated and may be more prone to misunderstandings. In a similar study, instead, women who delivered vaginally were less likely to receive appropriate IAP compared with those who delivered by cesarean section [9]. However, the authors excluded caesarian deliveries occurring less than 4 hours after rupture of membranes that were instead included in our study. Finally, the finding that appropriateness of IAP was significantly more likely in women who had no previous live birth may be related to a more careful management of first pregnancy prenatal care, although no association of IAP appropriateness with number of pregnancies was found in a previous study [9].

Several limitations should be acknowledged in this study. The study is retrospective and data were obtained by review of medical records and not directly observed, and documentation could have been not fully reported: indeed, we do not have information about GBS bacteriuria during current pregnancy and anamnesis for previous infants with EOGBSD diagnosis. Moreover, our data may not be representative of the entire region, reflecting practices of four maternity units. Finally, history of self-reported allergy to beta-lactam antibiotics was not documented by antimicrobial sensitivity testing, thus our results on this point and on appropriate agent choice for women with penicillin allergy were empirical.

### Conclusion

IAP is a success story for modern obstetrics, having decreased the rate of EOGBS infections by more than 80%. However, the findings of our study are in agreement with those of Verani et al. [35] that there is still room for improvement. Despite satisfactory GBS screening implementation, there is still a substantial gap between optimal and actual IAP implementation as first reported by Van Dyke et al. [15] and by Edwards et al. [30]. We hypothesize that the complexity of the CDC guidelines may partially explain this shortcoming. Future efforts will include initiatives focused at enabling and reinforcing adherence to evidence-based prevention practices. The efficacy of this strategy, as well as practices that diverged from best available evidence, should be evaluated systematically.

## Supporting Information

S1 File Dataset. This file reports the complete dataset to replicate the findings of the study.
(XLSX)
